# Digital models: How can dental arch form be verified chairside?

**DOI:** 10.1590/2177-6709.22.6.068-073.oar

**Published:** 2017

**Authors:** Alana Tavares, Emanuel Braga, Telma Martins de Araújo

**Affiliations:** 1 Universidade Federal da Bahia, Mestrado em Odontologia e Saúde (Salvador/BA, Brasil).; 2 Universidade Federal da Bahia, ​Departamento de Ortodontia (Salvador/BA, Brasil).

**Keywords:** Orthodontics, Dental arch, 3D imaging, Computer generated

## Abstract

**Introduction:**

Plaster dental casts are routinely used during clinical practice to access maxillary dental arch form and assist on fabrication of individualized orthodontic archwires. Recently introduced, digital model technology may offer a limitation for the obtainment of a dental physical record. In this context, a tool for dental arch form assessment for chairside use is necessary when employing digital models. In this regard, paper print of the dental arch seems thus to be useful.

**Methods:**

In the present study, 37 lower arch models were used. Intercanine and intermolar widths and dental arch length measurements were performed and compared using plaster dental casts, digital models and paper print image of the models. Ortho Insight 3D scanner was employed for model digitalization.

**Results:**

No statistically significant differences were noted regarding the measurements performed on the plaster or digital models (*p*> 0.05). Paper print images, however, showed subestimated values for intercanine and intermolar widths and overestimated values for dental arch length. Despite being statistically significant (*p*< 0.001), the differences were considered clinically negligible.

**Conclusion:**

The present study suggests that paper print images obtained from digital models are clinically accurate and can be used as a tool for dental arch form assessment for fabrication of individualized orthodontic archwires.

## RESUMO

**Introdução:**

os modelos de gesso são usados rotineiramente, durante a prática clínica, para avaliação da forma da arcada inferior e para auxiliar na confecção de arcos ortodônticos individualizados. A tecnologia dos modelos digitais, introduzida recentemente, pode oferecer uma limitação na obtenção de um registro físico da arcada dentária. Assim, quando se utilizam modelos digitais, faz-se necessária uma ferramenta clínica para obtenção da forma da arcada. Com essa finalidade, poderia-se imprimir, em papel, uma imagem da arcada dentária obtida a partir do modelo de gesso.

**Métodos:**

nesse estudo, 37 modelos da arcada inferior foram utilizados, nos quais foram realizadas medições das distâncias intercaninos, intermolares e comprimento da arcada; sendo, então, comparadas entre modelos de gesso, modelos digitalizados com um *scanner* Ortho Insight 3D e imagens impressas em folha de papel A4.

**Resultados:**

não foram encontradas diferenças estatisticamente significativas nas medidas realizadas nos modelos de gesso e modelos digitais (*p* > 0,05). As imagens impressas, contudo, mostraram valores subestimados para as distâncias intercaninos e intermolares, e superestimados para o comprimento da arcada. Apesar de serem estatisticamente significativas (*p*< 0,001), as diferenças foram consideradas clinicamente insignificantes.

**Conclusão:**

o presente estudo sugere que as imagens obtidas por meio dos modelos digitais e impressas em papel são clinicamente acuradas e podem ser utilizadas como uma ferramenta auxiliar na confecção dos arcos ortodônticos individualizados.

## INTRODUCTION

Plaster models are traditionally used as an essential part of the orthodontic documentation process.^1^ ^,^ ^2^ Combined to photographs, radiographs and clinical examination, plaster models provide important information for dental and skeletal malocclusions diagnosis and treatment.^3^

Plaster models are very convenient but indeed present disadvantages, such as need for significant physical space for storage, possible breakages or damages, microorganisms colonization in the long-term, possibility of loss, and difficulty to exchange information with other professionals.

Reducing physical files volume in dental offices is widely needed. In this context, digital records of patients have been increasingly incorporated in orthodontic offices. Digital models have recently been introduced in clinical orthodontics, having potential to replace plaster models and eliminate storage space issues.^4^ On the other hand, digital models also have some limitations, such as inability to be manually handled, need for software technical support and possible information loss. However, it is believed that such problems are less important compared to what digital technology may offer.^1^ ^,^ ^5^

In orthodontics, dental arch form maintenance is important, being directly related to function, aesthetics and stability.^6^ ^-^ ^8^ Therefore, arch shape, especially the maxillary arch, should not be altered throughout the treatment, in order to ensure outcome stability. 

It is common in clinical practice to use plaster models to assist in the preparation of individualized orthodontic archwires. In this sense, replacing plaster models by digital models implies the loss of a physical record to guide the orthodontist. Thereby, the present study aimed to test the fidelity of printed images obtained from the digitized model.

## MATERIAL AND METHODS

The study sample consisted of 37 lower dental arch plaster models depicting initial malocclusion of patients who undergone treatment in the Prof. José Édimo J. Soares Martins Orthodontics and Dentofacial Orthopedics Center, Federal University of Bahia (FOUFBA). The study was approved by FOUFBA ethics committee with the protocol number 35868414.5.0000.50.24. All participants signed an informed consent.

Patients were randomly selected and met the following inclusion criteria: complete permanent dentition up to first molars; no prosthetic restoration; and plaster models in perfect preparation and conservation state, without positive or negative bubbles or dental crown defects.

Evaluations were made in plaster models, digital models and printed images generated from digitized models. In order to evaluate plaster models, a Cen-Tech 4” (Harbor Freight Tools, Calabasas, CA, USA)digital caliper with 0.01mm accuracy and a specific plate made in CorelDRAW X5 containing two bold lines, one vertical and one horizontal, were used. Subsequently, a transparent adhesive was printed and fixed on a three millimeters thick glass plate, in order to keep the grid flat and facilitate assessments.

**Figure 1:**
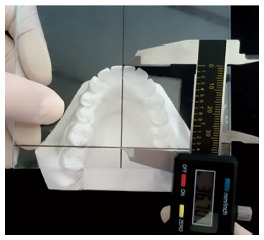
Plaster model arch length evaluation using the graduated plate and digital caliper.

Evaluation was performed considering the following measures:

» Arch length - measured in millimeters on a vertical line between lower central incisors to a horizontal line connecting the distal surfaces of teeth #36 and #46. A graduated plate specially designed for the study was used. The vertical line perpendicular to the horizontal line was positioned in a point between central incisors. Arch length was evaluated on the plate with a digital caliper (Fig 1). 

**Figure 2:**
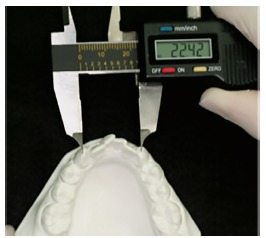
Plaster model intercanine width evaluation.

» Intercanine width - distance from tooth #33 cusp tip to tooth #43 cusp tip, measured in millimeters with a digital caliper (Fig 2).

**Figure 3:**
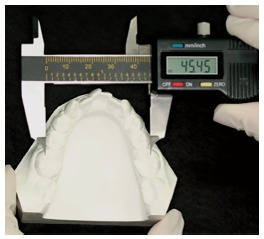
Plaster model intermolar width evaluation.

» Intermolar width - distance from tooth #36 mesiobuccal cusp tip to tooth #46 mesiobuccal cusp tip, measured in millimeters with a digital caliper (Fig 3).

**Figure 4:**
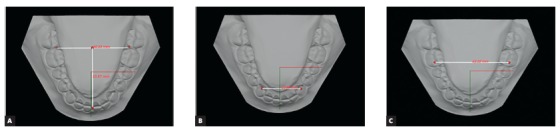
Digital model evaluation, as follows: A) arch length, B) intercanine and C) intermolar widths.

In order to evaluate digital models, lower arch virtual images were created from the plaster model through Ortho Insight 3D scanner, v. 5.0 (Motion View Software, LLC, Chattanooga, Tennessee, USA). Subsequently, arch length, and intercanine and intermolar widths measures were automatically generated by the program, following the same reference points used for the plaster model (Fig 4).

**Figure 5:**
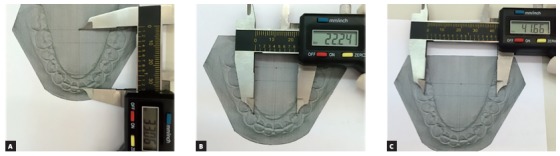
Printed image evaluation, as follows: A) arch length, B) intercanine and C) intermolar widths.

In order to evaluate the bi-dimensional (2D) image, the virtual models were printed in paper sheet directly from the software by selecting the capture 2D tool, clicking on printer and choosing the ABO 5 views option. Printing was conducted using a HP LaserJet 1020 printer (Hawlett - Packard Ltd., Campinas, SP, Brazil) set to letter size (279.4 x 215.9mm) with 100% original size and landscape orientation. On the printed image, arch length, intercanine and intermolar widths were measured with a digital caliper using the same reference points used for plaster and digital models (Fig 5).

Prior to measurements, in order to determine researcher calibration, five plaster models were randomly selected. Plaster model, digital model and printed image measurements were performed at two different times, two weeks apart, under the same conditions by the same researcher, which was properly trained. Measurements were subjected to statistical test to determine method error. For all variables, error was calculated according to Dahlberg’s formula, in order to verify intra-rater agreement. Measurement reproducibility analysis was conducted by intraclass correlation test. Both tests were set to 95% confidence level. The Bland-Altman test was also performed for measurement reproducibility analysis and the results were considered not clinically important.

Descriptive analysis was used to express the results as mean and standard deviation. To compare the dental arch measurements in the different methods, Variance Analysis for Repeated Measurements (ANOVA) was employed. Data distribution was evaluated with Shapiro-Wilk test. Significance level was set at 5% (α= 0,05). Data was tabulated and verified using IBM SPSS Statistics for Windows (IBM SPSS. 21.0, 2012, Armonk, NY: IBM Corp.).

## RESULTS

Table 1 shows the results for intercanine and intermolar widths and arch length, measured in the plaster models, digital models or paper prints. No statistically significant differences were noted regarding the measurements performed on the plaster or digital models (*p*> 0.05). Paper print images, however, showed subestimated values for intercanine and intermolar widths and overestimated values for dental arch length; these differences were considered statistically significant (*p*< 0.001) for the tested parameters. 

**Table 1:** Comparison between the different methods for evaluating intercanine and intermolar widths and arch length.

	Measures			p-valor
	Plaster Model	Digital Model	Print	
	Mean ± SD	Mean ± SD	Mean ± SD	
Intercanine width (mm)	26.14 ± 2.67a	26.11 ± 2.71a	25.73 ± 2.68^b^	< 0.001
Intermolar width (mm)	44.66 ± 3.40a	44.74 ± 3.43a	44.16 ± 3.41^b^	< 0.001
Arch length (mm)	33.94 ± 2.62a	33.99 ± 2.64a	34.42 ± 2.68^b^	< 0.001

a,b Horizontal values (line) with distinct letters indicate statistical difference (p < 0.05, comparisons between pairs with Bonferroni adjustment). Values are expressed as mean ± standard deviation.

## DISCUSSION

Literature reports that arch length, perimeter and incisors position change over time due to physiological reasons, regardless of orthodontic treatment.^9^ It is a consensus for most authors that arch shape and length should be preserved during orthodontic therapy, in order to achieve greater post-treatment stability.^6^ ^,^ ^7^ ^,^ ^10^ Plaster models have been the tool used to help orthodontists to reproduce individual dental arch shape of each patient during archwire bending. However, plaster models presents some disadvantages, such as need for significant physical space for storage, possible breakages and damage, possibility of loss and difficulty to exchange information with other professionals.^1^ ^,^ ^11^ In this context, three-dimensoinal (3D) scanned models have been proposed as a means to overcome plaster models limitations and facilitate orthodontics diagnose and planning.

As advantages, virtual models do not require physical space for storage, allow faster information exchange among professionals,^12^ and provide working options such as the virtual setup preparation.^13^ On the other hand, virtual models also have some limitations, such as the inability to be manually handled.^1^ Thus, it is necessary a mechanism to assist the orthodontist on accessing the arch shape when dealing with digital models.

The present study compared traditional plaster models, digitized models and paper prints obtained through the virtual models, aiming at providing a tool to help the orthodontist on accessing the dental arch shape in the daily clinical practice. No similar studies were found in the literature.

This study found no statistically significant difference (*p*> 0.05) comparing arch length, intercanine and intermolar width measurements between plaster and digital models. Different results were found by Santoro et al,^2^ but it was highlighted by the authors that the differences were within a clinical acceptable range and, thus acceptable for orthodontic use. Keating et al^11^ found no statistically significant differences between the two methods. Oliveira et al^14^ have also observed not statistically significant differences between methods, showing measurement reproducibility and reliability using digital models. Kim et al^15^ found excellent agreement between plaster and digitalized models, considering digitalized models reliable to replace traditional ones.

Paper print images, however, showed subestimated values for intercanine and intermolar widths and overestimated values for dental arch length. The differences were found to be statistically significant (*p*< 0.001). However, the comparison between the digital model and the paper print obtained from it, showed that for intercanine and intermolar widths the mean differences were 0.38mm and 0.58mm, respectively. Regarding arch length, the mean difference observed was -0.52mm. Previous published research,^2^ so as the authors of the present study, considered such differences as clinically negligible. It is thus suggested that the presented method is accurate for clinical use without bringing any potential distortions for the fabrication of orthodontic archwires or arch shape observation. 

It is important to note that the image was obtained according to the mentioned methodology and, therefore, must be reproduced. Different scanners may show different results, thus, proper studies for different manufacturers are recommended. 

Outcome stability, especially in relation to lower teeth irregularities, constitutes a key factor in orthodontic treatment. Several factors may contribute for increased stability and, among them, intercanine and intermolar widths maintenance are highlighted.^16^

According to accessed literature, arch shape change during orthodontic therapy potentiates relapse occurrence, making clear that, when possible, patient’s initial arch form is the best guide for future stability.^7^ ^,^ ^10^

The literature also shows evidence that intercanine and intermolar width decrease after treatment, especially if expansion was performed^17^. Glenn et al^18^ showed intercanine width and arch length decrease in the postretention period. Park et al^19^ have also found intercanine and intermolar width decrease after retainer removal. Moreover, Myser et al^20^ have also confirmed that there is intercanine width decrease after treatment, showing the importance of preserving the aforementioned distances and the arch shape.

Many researchers have sought methods for lower arch representation. A single and universal way may not represent the various features found in different individuals. Common arch shapes are simple to work with, yet challenging malocclusions may bring unusual arch forms.^21^ Therefore, in order to determine the arch shape of each patient, a reliable record is required, to preserve this shape during orthodontic therapy, thus contributing to treatment stability.

## CONCLUSIONS

The present study suggests that paper print images obtained from digital models are clinically accurate and can be used as a tool for dental arch form assessment for fabrication of individualized orthodontic archwires. 
